# Causes of maternal and child mortality among Cambodian sex workers and their children: a cross sectional study

**DOI:** 10.1186/s12889-016-3838-7

**Published:** 2016-11-21

**Authors:** Brian Willis, Saki Onda, Hanni Marie Stoklosa

**Affiliations:** 1 Global Health Promise, 2434 SW Sherwood Dr, 97201 Portland, OR USA; 2 Department of Obstetrics and Gynecology, Bridgeport Hospital – Yale New Haven Health, 267 Grant St, 06460 Bridgeport, CT USA; 3 Department of Emergency Medicine, Brigham and Women’s Hospital, 75 Francis St, 02115 Boston, MA USA

**Keywords:** Sex workers, Children of sex workers, Maternal mortality, Infant mortality

## Abstract

**Background:**

To reach global and national goals for maternal and child mortality, countries must identify vulnerable populations, which includes sex workers and their children. The objective of this study was to identify and describe maternal deaths of female sex workers in Cambodia and causes of death among their children.

**Methods:**

A convenience sample of female sex workers were recruited by local NGOs that provide support to sex workers. We modified the maternal mortality section of the 2010 Cambodia Demographic and Health Survey and collected reports of all deaths of female sex workers. For each death we ask the ‘sisterhood’ methodology questions to identify maternal deaths. For child deaths we asked each mother who reported the death of a child about the cause of death. We also asked all participants about the cause of deaths of children of other female sex workers.

**Results:**

We interviewed 271 female sex workers in the four largest Cambodian cities between May and September 2013. Participants reported 32 deaths of other female sex workers that met criteria for maternal death. The most common reported causes of maternal deaths were abortion (*n* = 13;40%) and HIV (*n* = 5;16%). Participants report deaths of 8 of their children and 50 deaths of children of other female sex workers. HIV was the reported cause of death for 13 (36%) children under age five.

**Conclusion:**

This is the first report of maternal deaths of sex workers in Cambodia or any other country. This modification of the sisterhood methodology has not been validated and did not allow us to calculate maternal mortality rates so the results are not generalizable, however these deaths may represent unrecognized maternal deaths in Cambodia. The results also indicate that children of sex workers in Cambodia are at risk of HIV and may not be accessing treatment. These issues require additional studies but in the meantime we must assure that sex workers in Cambodia and their children have access to quality health services.

## Background

Preventing maternal deaths is a global priority. Millennium Development Goal (MDG) 5A established the goal of reducing the maternal mortality ratio (MMR) by 75% by 2015. As of 2013, the MMR had decreased globally 47%, from 400 to 210 maternal deaths per 100,000 live births, and in 2013 there were 289,000 maternal deaths, 50% fewer deaths than in 1990 [1, 2]. To continue to reduce the number of maternal deaths under the Sustainable Development Goals (SDG) we need to identify both the causes of maternal deaths and also which women are most- at-risk for maternal deaths so that effective interventions can be targeted to those women.

In Cambodia the MMR decreased from 432 to 206 maternal deaths per 100,000 live births between 2000 and 2010 and to 170/100,000 in 2013 [3, 4]. In 2009 the Cambodian Ministry of Health mandated reporting and investigation of all maternal deaths. However, a 2012 study determined that only 20–30% of all maternal deaths in Cambodia are officially reported [5]. The 2012 study also reported 77% of all maternal deaths in Cambodia were due to direct causes with hemorrhage resulting in 36% of maternal deaths. In 2015, the MMR was 161/100,000 and it was determined that, unlike many developing countries, Cambodia had successfully achieved its Millennium Development Goal 5A [6]. Cambodia has also been able to effectively reduce its MMR below the global MMR of 216/100,000 and that of its neighbor, Laos (197/100,000) but it is still above the MMR for Thailand and Vietnam (54/100,000 and 20/100,000 respectively) [7].

Although decreasing maternal deaths is a priority, these deaths are difficult to identify as they are relatively rare [8]. This makes identifying maternal deaths difficult and even more challenging in subpopulations of vulnerable and marginalized women, including female sex workers (FSW). Yet identifying maternal deaths among FSW is critical to national and global efforts to prevent maternal deaths as health and structural vulnerabilities of FSW may also increase their risk of maternal mortality. Identifying maternal deaths among FSW may require new and innovative methodologies or modifications of existing methodologies.

Although FSW comprise a small percentage of all women, according to UNAIDS there are “tens of millions” of sex workers globally [9]. Millions of FSW have vulnerabilities directly related to their work and home environments that increase their risk of serious health problems, including HIV and violence [10, 11]. Just as FSW are among the most-at-risk population for HIV, they may also be among the most-at-risk women for maternal mortality due to their underlying health problems, including poor nutrition and TB, and vulnerabilities. It is therefore critical to determine if, as with HIV, FSW are also at high risk for maternal mortality.

Similarly, a very large percentage of FSW have children but no data on the causes of death among their children has been identified. As with FSW, their children may constitute a small percentage of all children globally but there are likely to be many millions of children of FSW who, like their mothers, have risk factors that significantly increase their risk of mortality. It is important to study mortality among children of FSW in order to determine if they have higher mortality or different causes of death than other children of similar socio-economic situations. If they do have higher mortality than other children, or different causes of death, appropriate prevention programs and policies can be implemented to prevent these deaths.

Regarding FSW in Cambodia, country specific studies document their HIV rates ranging from 9.2% to 13.9% [12, 13]. FSW also have high rates of other sexually transmitted infections (STIs) and substance abuse [14]. Over a quarter (28.2%) of FSW in Cambodia have had an abortion but there are no data on abortion-related mortality among FSW [15].

Globally, there are few studies on children of FSW [16]. Two studies report deaths among these children, ranging from 7–16%, but neither reported the causes of death [17, 18]. While the causes of death of children in Cambodia have been identified (Table 3), there are no data on causes of death among children of FSW and it is unknown if they have the same or different causes of death as all other children in Cambodia.

## Methods

In order to identify maternal mortality of FSW in Cambodia and causes of deaths of their children we conducted a survey of FSW using components of the Women’s Questionnaire section from the 2010 Cambodian Demographic Health Survey (CDHS). In order to identify maternal deaths among FSW in Cambodia we modified section 11, Maternal Mortality, which utilizes the sisterhood methodology. The sisterhood methodology was developed to measure maternal mortality in countries where death registries are incomplete and relies on the informed knowledge of deaths based on kinship, particularly sisters within a household. FSW in Cambodia may not live with their families or biological sisters so the sisterhood methodology, based on reports from sisters within a household, may not identify maternal deaths of FSW. Rather, in this study, FSW were considered surrogate sisters of other FSW based on their strong social networks.

The sisterhood methodology is used in some national Demographic Health Surveys (DHS), including Cambodia, to identify maternal deaths during household surveys. In the 2010 CDHS 40 maternal deaths were identified during interviews with 18,754 women via the sisterhood methodology. The 2010 CDHS defined maternal deaths “as any death that occurred during pregnancy, childbirth, or within 6 weeks after the birth or termination of a pregnancy….even if the death is due to causes that are not pregnancy related.” We used the same definition in this study. The 2010 CDHS did not identify causes of maternal deaths so it is not possible to determine which were due to direct and indirect causes.

In this study we asked participants for information on all FSW they knew who had died. For every reported death of a FSW additional information on the deceased woman was collected, including: first name, age at death, date of death, and the city where she died, cause of death, number of children she had, and whether the deceased FSW died while pregnant, during childbirth, or within six weeks after the end of a pregnancy or childbirth. Here we report only the deaths of FSW that occurred during pregnancy, childbirth, or the postpartum period. The data on all other deaths of FSW will be reported in another paper.

Similar to the recall period used in the 2010 CDHS, only maternal deaths reported during the seven years preceding this study, e.g., 2007–2013, the “look back” period, were included in our analysis.

To identify causes of deaths of children of FSW, two methods were utilized. First, for deaths of children of the participants we used Methods, Reproduction, from the Women’s Questionnaire of the 2010 CDHS form that records information on the children of all participants, including date of birth, gender, and, if deceased, age of death. To these questions we added an additional question on the cause of death, which was only asked when participants reported the death of a child. In the second method, we developed a list of questions in which all participants were asked if they knew of the death of the death of the child of another FSW. As with reports of maternal deaths of FSW based in the social network where FSW know of the deaths of other FSW, they often also know about the deaths of children of other FSW. Therefore participants were asked to report the deaths of children of other FSW along with specific information to permit identification of potential duplicate reports of deaths of children of FSW, e.g., the name of the deceased child, year of death, gender, age at death, and cause of death. Only child deaths that occurred within the past six years (2008–2013), the “look back” period for this study, were analyzed. This is the same number of years used by the 2010 CDHS as the “look back” period for child deaths. While the 2010 CDHS reported the number of child deaths that were reported, it did not report the causes of these deaths.

All FSW in the study were at least 18 years-old, had been in sex work for at least three years, had been pregnant at least once while in sex work, had engaged in sex work with at least two men during the past year, and spoke Khmer. The inclusion criterion for minimum number of clients of FSW in the past year was intentionally low to account for the period of time they did not work if they were pregnant and the postpartum period.

### Sample size and sampling procedure

In 2012, a year before this study was conducted, a national census of FSW in Cambodia, identified 7,498 FSW throughout the country. Using the 2012 census with a 5% margin of error and a 90% confidence interval the sample size for this study was 262 FSW. Interviews of FSW were conducted in the four largest cities in Cambodia: Phnom Penh, Sihanoukville, Battambang, and Siem Reap. FSW were identified via purposive sampling in which field-based peer educators informed FSW in their respective geographic area about the study and the inclusion criteria and specifically informed any FSW who met the inclusion criteria about the study. In three of the cities, FSW who chose to participate took local transportation to the NGO office, but in Battambang the NGO had outreach workers with motorcycles bring the FSW due to limited public transportation. A total of 271 FSW participated in the study with 67–69 FSW interviewed in each city. Interviews were conducted between May and September 2013.

### Data management, quality control, and data analysis

Interviewers included staff from the NGOs that partnered on the study in Siem Reap and Battambang. In Sihanoukville and Phnom Penh three university students were recruited as interviewers. All interviewers were trained by the PI and co-PI on the procedure for reading the information sheet and completing the consent forms, compensation, and entering data on the questionnaire. All staff had previous experience working with FSW and collecting data. Due to the sensitive nature of some of the questions, only females were recruited as interviewers. The PI or co-PI observed interviews by each interviewer before they were permitted to conduct unsupervised interviews. Completed questionnaires were reviewed by the PI for completeness before the FSW left the NGO office. Interviews were anonymous so each questionnaire was given a code based on the city where it was collected and the numerical sequence in which participant were interviewed, e.g., questionnaires in Phnom Penh were coded “PP-1” to “PP-67.”

Completed questionnaires and consent forms were all kept in a locked and secured file cabinet at University of Health Science (UHS), the national partner for the study, by the PI. Digital Data Divide, an NGO in Cambodia, was contracted to enter data from the questionnaires into Epi-Info Version 7 (Centers for Disease Control). Cases of maternal and child deaths were extracted into Microsoft Excel. Duplicates deaths were identified by the PI and co-PI and deleted. Frequency tables with demographic information, year of death, city of death, number of children, and cause of death were created.

### Ethical considerations

The Cambodian National Ethics Committee for Health Research (NECHR) is responsible for all studies involving human subjects in Cambodia. The application to the NECHR for a study requires information sheets about the study for each participant, along with informed consent forms, in both English and Khmer. A commercial translation service translated the information sheets and consent forms from English to Khmer, which were reviewed by the Cambodian co-PI.

The information sheets and consent form informed all participants that they did not have to participate in the study, did not have to answer any question they did not want to, and could terminate the interview at any time and still keep their compensation.

The application for the study was reviewed and approved by UHS. It was then submitted to the NECHR. In addition, each partner NGO reviewed and approved the questionnaires and interviewers. The information form and consent were read to the participants in Khmer by the interviewers, who were all Cambodian and native Khmer speakers. After oral consent was confirmed each participant received a copy of the consent form and compensation. Every participant received the equivalent of US$5 for participation and US$2.50 for transportation expenses, except in Battambang where peer educators were compensated for transporting the women to the NGO office.

Meals and refreshments were also provided for all the participants and their children and childcare was offered for the mothers who brought their children with them. Interviews took 30–45 min and were conducted in rooms at the NGOs offices that provided privacy and protected the confidentiality of the participants.

The data analysis by the co-authors was approved by the Harvard School of Public Health and Partners Healthcare Institutional Review Board.

## Results

A total of 271 eligible FSW participated in the study. The age of the women ranged from 18–45 years (mean 28.5 ± 5.4) and had been a FSW an average of 7.1 years (SD ±4.0). The majority were ethnically Khmer (252; 92.9%) and the remainder were Cham, Vietnamese, or mixed ethnicity. Most of the women (220; 81.4%) had some education but was limited to primary school for the majority (102; 73.6%) (Table 1).

**Table 1:** Demographic characteristics of study group and the 2010 Cambodia Demographic and Health Survey (CHDS)

		FSW (*n* = 271)	CDHS (*n* = 18,754)
Age (mean, range)		28.5 (18–45)	(15–49)
Years as FSW (mean)		7.2	–
Level of education (%)	No schooling	18.5	15.9
	Primary	59.8	49.4
	Secondary or higher	21.4	34.7
Parity (mean, range)		2.0 (1–6)	NA

Many FSW reported working in multiple locations (total responses 324; 119.5%), including karaoke bars (140; 51.6%), beer gardens (32; 11.8%), bars (47; 17.3%), massage parlors (47; 17.3%), and other venues (3; 1.1%). All of the women had been pregnant at least once since 2008 and 30 (11.0%) were currently pregnant. The majority of the women (210; 77.4%) had given birth at least once since 2008.

A total of 194 deaths of FSW were reported by 132 (48.7%) of the participants. After independent review by the PI and co-PI reported deaths were excluded if they matched another death on two or more demographic factors. Among all the reported deaths, nine were excluded as possible duplicates but none of these deaths included any of the reported maternal deaths.

Among the reported deaths, 43 (22.1%) occurred after the FSW had an abortion, during childbirth, or within six weeks of termination of a pregnancy or childbirth. Of these 43 deaths, we analyzed 32 that occurred within the past seven years, which was the same number of years used in the 2010 CDHS to identify maternal deaths in Cambodia. The majority of these deaths (24; 75%) were reported in the four cities where interviews were conducted, with the most reported in Phnom Penh (8; 25.0%) and the fewest in Sihanoukville (3; 9.0%). The deceased FSW ranged in age from 17–42 years old (mean 26.5) and 15 (48.8%) had 1–4 children at the time of their deaths. The most common causes of deaths among these 32 FSW was abortion (*n* = 13;41%) and HIV (*n* = 5;16%). Other causes are listed in Table 2.

**Table 2:** Causes of maternal deaths

Causes of maternal deaths among sex workers (*N* = 32)			Causes of maternal deaths in Cambodia^a^ (*N* = 153)
Abortion	13	(41%)	6 (4%)
HIV	5	(16%)	–
Obstructed labor	3	(9%)	–
Suicide	3	(9%)	–
Accident	2	(6%)	–
Murder	1	(3%)	–
Postpartum hemorrhage	1	(3%)	55 (36%)
Unknown	4	(13%)	–
Other direct causes		–	32 (21%)
Pre-eclampsia		–	30 (20%)
Sepsis		–	12 (8%)
Heart disease		–	11 (7%)
Uterine rupture		–	5 (3%)
Anemia		–	2 (1%)

^a^Based on unpublished data from the Cambodian National Maternal and Child Health Centre of maternal deaths in 2010 reported by Liljestrand

Mothers reported giving birth to 469 children, of which 14 (2.9%) died, 8 (57.1%) of which occurred between 2008–2013, our “look back” period. All eight of these children were 18 months old or younger and 5 (35.7%) were a month-old or younger.

In three of the cities, 46 (26.2%) of 175 participants reported 67 deaths of a child of another FSW between 2001 and 2013. (This question was inadvertently excluded during the interviews in the first city, Siem Reap.) We excluded seven deaths for which the year or age at death was unknown, one possible duplicate report, and nine deaths that occurred outside of our “look back” period of 2008–2013, resulting a total of 50 child deaths. Of these children, 26 (52.0%) were male and 24 (48.0%) were female. The age range for deaths was <1 day to 10 years, with a mean of 2.9 years. The most commonly reported cause of death was HIV (14/28%), followed by lung disease (8/16%), and fever (6/12%). Other causes of death included dengue, drowning, liver disease, and measles. For 10 (20%) of the deaths participants reported the cause of death was unknown.

## Discussion

Previous studies of FSW in Cambodia have not included data on their maternal deaths or deaths of their children. This is the first known study to identify maternal deaths of FSW in Cambodia or any other country and causes of death among children of FSW in Cambodia.

Maternal deaths among FSW are not explicitly recognized under the existing system for identifying maternal deaths in Cambodia. Regardless, as this study suggests, there may be many unrecognized maternal deaths among FSW in Cambodia. Based on the high proportion of these deaths related to abortion, they may indicate FSW have barriers to reproductive health and safe abortion, use unsafe methods of abortions, and experience barriers to post-abortion care. In addition, based on the maternal deaths due to HIV in our study, HIV-infected FSW may represent a very large but unrecognized population of women at risk of maternal mortality in Cambodia and other countries where FSW have high rates of HIV but limited access to HIV treatment [19].

For two perspectives on our findings, the 2010 CDHS identified 40 maternal deaths among the general population through interviewing 18,754 women while we identified 32 maternal deaths through interviewing a much small sample of 271 FSW. In addition, the leading causes of deaths were different for FSW and other women. In the 2012 analysis of maternal deaths in Cambodia, 77% were due to direct causes, of which hemorrhage resulted in 55 (36%) deaths and abortion resulted in 6 (4%) maternal deaths. (Table 2) In our study, 58% of maternal deaths among FSW were due to direct causes: 13 (40%) were due to abortion, 3 (9%) from obstructed labor and 3 (9%) postpartum hemorrhage. Indirect causes results in 42% of the maternal deaths with HIV (5/16%) resulted in the most deaths from indirect causes. Of the abortion-related deaths in our study, 9 (69.2%) occurred between January 2011 and August 2013 and represent recent deaths.

It is unclear what impact unrecognized maternal deaths among FSW would have on the MMR in Cambodia or successfully achieving MDG 5A. Going forward, however, the risk of maternal deaths among FSW should be considered as the SDG are implemented.

Similarly, no study reporting the causes of death of children of FSW have been identified from Cambodia or other countries. This lack of data on children of FSW, including causes of mortality, compromised our ability to know if they are receiving the services they need. With specific data on children of FSW we will be able to develop evidence-based interventions base on local or national data. For example, in sub-Saharan Africa the children may be at risk of death from HIV but experience barriers to treatment. Similarly, in developed countries the children may have more complications from exposure to illegal substances, such as heroin, in utero. In both of these situations, data on the health of these children and barriers to health services will allow local interventions to be developed to address the unmet needs of these children.

The 14 child deaths reported by FSW represent 2.9% of all live births among participants and may reflect under-reporting as the child death questions were not ask in one city and, as the 2010 CDHS notes, due to a cultural issue some Cambodian mothers do not want to discuss the death of their own children. Regardless, we note that the majority of these deaths (13; 92.8%) occurred in children less than 18-months-old. This may indicate a particularly high risk period for infant of FSW.

This is the first known use of the sisterhood methodology to identify maternal deaths of FSW and has not been validated for this use. However, based on the validity of both the motherhood and neighborhood methodologies to reliably report maternal and infant deaths by persons other than family members, we believe the sisterhood methodology can also be reliably used to identify maternal deaths of FSW and FSW can reliably report the deaths of children of other FSW [20, 21].

Another method that can be used to identify maternal deaths among FSW and deaths of their children is the Verbal Autopsy (VA) in which the cause of death is ascertain based on an interview with a family member or caregiver [22].

It is possible that FSW in our study did not report all the maternal deaths of FSW or their children or accurately recall the details of these deaths. It is also possible that some of these deaths could be duplicative but the details about each death allowed us to mitigate this possibility by excluding potentially duplicative reports. Due to lack of resources, we could not compare reported maternal deaths of FSW with officially recorded maternal deaths so it is unknown if these deaths had been reported to health authorities.

Among the reported deaths of children of other FSW in our study, most importantly perhaps is the large number of deaths attributed to HIV. Since FSW have higher rates of HIV than do other women but often have less access to HIV prevention and treatment, it is not unexpected that many of their children would also be infected with HIV. However HIV among children of FSW is not reported among the causes of death among children in Cambodia (Table 3) or in the literature on pediatric HIV. The participants did not provide any other additional information about HIV-related deaths, e.g., whether the death was due to an AIDS-related condition, but additional details should be elicited in future studies.

**Table 3:** Causes of death among children in Cambodia

Children of other sex workers (FSW) under 5 years of age (*N* = 36)		Children of other FSW over 5 years of age (*N* = 14)		Children of FSW under 5 years of age (*N* = 8)		Children in Cambodia overall^a^	
HIV	13 (36.1%)	HIV	1 (7.1%)	–		HIV/AIDS	0.30%
Lung disease	6 (16.7%)	Lung disease	1 (7.1%)	Pneumonia	1 (12.5%)	Acute lower respiratory infections	16.80%
Dengue	3 (8.3%)	Dengue	2 (14.3%)	–		–	
Fever	2 (5.6%)	Fever	2 (14.3%)	Fever	2 (25%)	Sepsis and other infectious conditions of the newborn	8.10%
Drowning	2 (5.6%)	Drowning	2 (14.3%)	–		–	
Hepatitis B	3 (2.8%)	Liver disease	1 (7.1%)	–		–	
Congenital heart disease	3 (2.8%)	–		–		Congenital anomalies	9.80%
Measles	3 (2.8%)	–		Measles	1 (12.5%)	Measles	0.40%
Malnutrition	3 (2.8%)	–		–		–	
Unknown	6 (16.7%)	Unknown	2 (14.3%)	Unknown	2 (25%)	–	
–		Road accident	3 (21.4%)	–		Injuries	8.30%
–		–		Diarrhea	1 (12.5%)	Diarrheal diseases	8.10%
–		–		Asthma	1 (12.5%)	–	
–		–		–		Prematurity	16.20%
–		–		–		Birth asphyxia and birth trauma	13%
–		–		–		Other communicable, perinatal and nutritional conditions	9.40%
–		–		–		Other non-communicable diseases	4.50%
–		–		–		Pertussis	2.80%
–		–		–		Meningitis/encephalitis	1.40%
–		–		–		Malaria	0.50%
–		–		–		Tetanus	0.304

*WHO* Global Health Observatory. Causes of child mortality. http://www.who.int/gho/child_health/mortality/causes/en/

Building on this study, we must better understand the risks of maternal mortality, as well as maternal morbidity, among FSW in Cambodia. This will require additional studies to identify factors related to sex work, including factors that increase their risk of HIV. For example, we must understand if FSW who are mothers engage in more high risk sex compared to FSW who do not have children in order to feed their children and if there are barriers to reproductive health and safe abortion that other Cambodian women do not experience. Likewise we must understand if children of FSW in Cambodia have more or different risks to their health compared to other vulnerable children that result in higher mortality rates. The data on both maternal and child deaths are needed to determine if additional programs and policies for FSW and their children in Cambodia are needed.

The sample size for this study was small and was limited to four cities with Khmer-speaking FSW. This may have resulted in the exclusion of FSW who only speak other languages, especially Vietnamese, and the deaths reported from these four cities may not be representative of maternal deaths of FSW and their children throughout Cambodia. Without a denominator it was not possible to calculate the MMR and the data on children were too small to calculate mortality rates. The lack of data on deaths of children of other FSW in Siem Reap also likely resulted in the reporting of fewer child deaths.

A better understanding of the maternal health of FSW and the health of their children, including why they die, is critical in Cambodia and other countries.

## Conclusion

To achieve goals for maternal and child health in Cambodia and other countries it is critical to reach the most vulnerable and marginalized mothers and their children, including FSW and their children. Although there are many studies on FSW in Cambodia and globally, their risk for maternal morbidity has received inadequate attention. The modified sisterhood methodology piloted in this study should be evaluated for use in other countries along with other methodologies, such as the motherhood methodology, to identify maternal deaths among FSW. These studies are needed to identify unrecognized health problems or barriers to health services that may result in high maternal mortality among FSW compared to other women.

Likewise we need data on the health of millions of children of FSW in Cambodia and other countries, including their causes of death, to understand if they experience more or different health problems than other children and if they die from causes different than those of other children. Specifically we need studies to understand if a higher percentage of children of FSW are infected with HIV than other children and, if so, why they have higher rates.

In addition to studies, routine surveillance systems to record deaths of FSW and their children should be developed. Such systems could facilitate data to be collected and analyzed annually, which would allow FSW, sex worker NGOs, and NGOs and government agencies that provide support to FSW and their children to identify trends and use data to implement and modify services as needed. The use of standardized data collection in countries would allow data on deaths of FSW and their children to be aggregated and used for regional and global analysis as is done for HIV.

This study highlights the potential risk to FSW and their children in Cambodia and other countries from maternal mortality and preventable causes of childhood deaths. While additional studies are needed to confirm these results we should also ensure that FSW and their children have access to reproductive health, quality prenatal care and delivery, and child health services.

## Abbreviations

MDG: Millennium development goal
MMR: Maternal mortality ratio
SDG: Sustainable development goals
FSW: Female sex workers
MTCT: Maternal-to-child-transmission
CDHS: Cambodian Demographic Health Surveys
STIs: Sexually transmitted infections
DHS: Demographic health surveys
